# Validity and reliability of the Turkish version of the Regret Intensity Scale

**DOI:** 10.1186/s12912-025-03992-z

**Published:** 2025-10-28

**Authors:** Ela Yılmaz Coşkun, Şengül Üzen Cura, Aysel Özsaban

**Affiliations:** 1 Department of Nursing, Faculty of Health Sciences, Tekirdağ Namik Kemal University, Campus Street, Number: 1, Tekirdağ, 59030 Türkiye; 2 Department of Nursing, Faculty of Health Sciences, Canakkale Onsekiz Mart University, Terzioğlu Campus, Canakkale, Türkiye; 3 Department of Fundamentals of Nursing, Faculty of Health Sciences, Karadeniz Technical University, University Neighbourhood, Farabi Street, Number: 88, Ortahisar / Trabzon, 61080 Türkiye

**Keywords:** Care, Nursing, Validity, Reliability, Regret, Scale

## Abstract

**Aims:**

This study aimed to evaluate the validity and reliability of the Turkish version of the Regret Intensity Scale, which assesses the regret experienced by nurses during the care process.

**Methods:**

This methodological study was conducted with 402 nurses. Data were collected using the Nurse Information Form, Regret Intensity Scale and Elements of Regret Scale. Language and content validity were assessed using the Content Validity Index. Construct validity was examined through exploratory and confirmatory factor analyses. Cronbach’s alpha coefficient, item-total score correlation, and Pearson correlation coefficient were used to assess reliability.

**Results:**

Exploratory factor analysis (*n* = 193) showed a KMO value of 0.890 and a Bartlett’s test value of 1466.942 (*p* < 0.001). Factor loadings of the single-factor structure ranged from 0.737 to 0.865. Confirmatory factor analysis (*n* = 209) indicated good model fit (χ2/df = 2.338, RMSEA = 0.079, GFI = 0.95). The Cronbach’s alpha coefficient of the scale was 0.932 and all items ranged from 0.927 to 0.934. A statistically significant correlation was found with the Elements of Regret Scale (*r* = 0.490; *p* < 0.001).

**Conclusion:**

The Turkish version of the Regret Intensity Scale is a valid and reliable instrument for measuring the regret experienced by nurses in care processes. It is a practical tool for use in clinical practice and research to assess care-related regret in Turkish-speaking healthcare professionals.

## Introduction

Regret is a negative emotion experienced when individuals believe that a better outcome could have been achieved by choosing a different course of action [1–3]. In healthcare, regret develops when professionals feel that they have failed to provide what they consider to be optimal care for their patients [4, 5]. Nurses today increasingly provide care to complicated patients with multiple comorbidities, many of whom are elderly and from diverse cultural backgrounds [6]. Challenges such as patient suffering, end-of-life care, and conflicting care needs may contribute to feelings of regret [7–9]. In a study conducted in Switzerland, the prevalence of regret was reported to be 15% among nurses and 10% among physicians [6].

Nurses are particularly prone to regret because they face heavy emotional burdens and rapid decision-making processes [10]. Care-related regret refers to the feeling of regret that nurses experience as a result of the decisions they make about patient care or the care processes they deliver [11]. This may occur when outcomes are unsatisfactory, complications arise, or decisions conflict with ethical or professional values [3]. Unmet expectations of patients or family members, limited resources, or insufficient professional knowledge are among the main triggers [5, 6]. Importantly, regret not only influences decision-making but may also promote learning and practice improvement for future care [9, 10, 12]. Studies have shown associations between care-related regret and negative outcomes such as sleep disturbances, burnout, and lower job satisfaction [13–17]. Measuring care-related regret is therefore essential [3].

Although regret can be conceptually measured based on existing literature, there is a lack of valid and reliable instruments to measure care-related regret among nurses. Such instruments should be both reliable and brief, making them suitable for regular monitoring [6]. The Regret Intensity Scale, developed by Courvoisier et al., is the most widely used instrument for this purpose [5]. The scale has been translated into German [6] and Portuguese [18], and applied to various health professionals (nurses, physicians, physiotherapists, etc.). It has also been validated among nursing interns in China [8]. However, no study has yet examined care-related regret in Türkiye, and no validated instrument exists for this population.

This study aimed to adapt and validate the Turkish version of Courvoisier et al.’s Regret Intensity Scale (RIS-10), rather than develop a new instrument. The RIS-10 was selected because it is a brief, validated, and widely used measure that directly captures the intensity of regret rather than coping strategies [9]. This focus aligns with our objective of quantifying the emotional burden of regret among nurses. Compared to broader tools such as the Regret Coping Scale, which evaluates coping mechanisms, the RIS-10 provides a more precise and practical assessment of regret intensity [6, 9]. The primary contribution of this study is the establishment of cross-cultural validity and the introduction of a reliable instrument into the Turkish context that facilitates international comparability.

## Materials and methods

### Study type and purpose

This methodological study was conducted to establish the Turkish validity and reliability of the Regret Intensity Scale.

#### The research questions were as follows

* Is the Regret Intensity Scale a valid measurement tool in evaluating nurses’ care-related regret?

* Is the Regret Intensity Scale a reliable measurement tool in evaluating nurses’ care-related regret?

##### Population and sample

This study was conducted with nurses working in public hospitals in Türkiye. For scale validity and reliability studies, it is recommended that the sample size should be at least 10 times the number of scale items to perform factor analysis and 20 times the number of scale items to create a more robust structure in the scale [19, 20]. In addition, a sample size of at least 200 should be used for confirmatory factor analysis [21]. A larger sample size has also been suggested in the relevant literature [19]. Accordingly, 402 nurses were reached in this study. The recruitment process was carried out electronically. Notices were distributed through hospital nursing directorates, institutional mailing lists, and professional WhatsApp groups, inviting eligible nurses to participate. Interested nurses accessed the study via a secure Google Forms link. Participation was entirely voluntary, and no incentives were provided. The inclusion criteria were being a nurse, having responsibility for patient care and providing consent to participate in the study.

### Data collection tools

#### Nurse information form

It is an eight-item form prepared by the researchers in line with the literature [5, 18]. The form asks nurses about their age, gender, education level, years of experience, service type, shift type, number of patients cared for, and experience with regret regarding care.

#### Regret intensity scale

The scale for measuring health professionals’ care-related regret was developed by Courvoisier et al. in Switzerland [5]. The scale is unidimensional and consists of 10 items covering symptoms of regret in emotional, physical, and cognitive domains. Participants rate each item on a 5-point Likert scale ranging from 1 (strongly disagree) to 5 (strongly agree). The total score ranges from 10 to 50, with higher scores indicating more intense care-related regret. The original scale has a Cronbach α coefficient of 0.87 and test-retest reliability of 0.70 [5]. For this study, Cronbach’s α was 0.932; subscale values ranged from 0.928 to 0.934.

#### Elements of regret scale

This instrument, developed by Buchanan et al. [22], distinguishes the *emotional* and *cognitive* components of regret. The scale consists of 10 items, with the first five assessing emotional regret and the last five assessing cognitive regret. It has been adapted into Turkish by Aktu [1], showing good psychometric properties, which makes it an appropriate comparator for evaluating the construct validity of the RIS-10 in this study. Scores between 10 and 70 can be obtained from the scale. The higher the scores, the more regret one feels about life [1]. The Cronbach alpha reliability coefficient was 0.94 in the original version of the scale [22], 0.92 in the Turkish version [1], and 0.96 in this study.

#### Data collection

Data were collected electronically using Google Forms between August and December 2024. Nurses who met the inclusion criteria could access the online questionnaire through the distributed link. Participants first viewed an informed consent page, which explained the study purpose, confidentiality measures, and the voluntary nature of participation. Only those who actively checked the box stating “I voluntarily agree to participate in this research” were able to proceed to the survey questions. The questionnaire included the Nurse Information Form, the Regret Intensity Scale, and the Elements of Regret Scale. To minimize duplicate submissions, the Google Form was set to allow only one response per device. Survey completion took approximately 15–20 min. A total of 402 valid responses were obtained after excluding incomplete or inconsistent submissions.

#### Data analysis

Statistical analyses were conducted using SPSS (v.26.0), LISREL (v.8.70), and RStudio (v.2023.09.1.494). SPSS was used for descriptive statistics and preliminary reliability analyses, as it is widely applied in health sciences research. LISREL was employed for confirmatory factor analysis (CFA), given its strong capability for structural equation modeling and goodness-of-fit testing. RStudio was used to support advanced data handling and reproducibility of statistical procedures, in line with current recommendations for transparency in psychometric research [19, 21].

Descriptive data were presented as frequencies, means, standard deviations, and percentages. Content Validity Index (CVI) was used for the language and content validity of the scale. For construct validity, the total sample of 402 nurses was randomly divided into two independent groups. Data from 193 participants were used for the exploratory factor analysis (EFA), while data from 209 participants were used for the confirmatory factor analysis (CFA). Randomly splitting the dataset into subsamples is recommended in methodological literature to strengthen construct validation and minimize the risk of model overfitting [19, 21]. EFA with Varimax rotation was conducted to explore the factor structure, while CFA was performed to verify the model fit. The adequacy of the data for factor analysis was assessed using the Kaiser–Meyer–Olkin (KMO) measure and Bartlett’s test of sphericity. Reliability was evaluated through Cronbach’s alpha coefficient for internal consistency, item–total score correlations, and parallel form validity using Pearson’s correlation coefficient.

## Findings

The nurses who participated in the study had a mean age of 30.68 ± 7.99 years. Of the participants, 83.6% were female, 64.2% held undergraduate degrees, and 29.9% had 10 years or more of professional experience. 45.3% of the nurses worked in inpatient wards, 68.2% worked in shifts, and cared for an average of 13.03 ± 50.79 patients in each shift. 67.2% of them reported having ever experienced care-related regret.

### Findings related to validity

#### Content validity

The content validity of the scale, which was translated and back-translated, was evaluated by 10 nursing experts holding doctoral degrees. Davis’ technique was utilized to determine the content validity. According to the Davis technique, the scale was examined on a scale of 1–4 (1 = inappropriate, 2 = somewhat appropriate, 3 = quite appropriate, and 4 = very appropriate) to determine that each item was clear, meaningful, and unambiguously and linguistically correct. At least 80% of the experts were expected to rate the scale items as “quite” or “very” appropriate [23]. After each item was rated, the number of experts who marked option 3 or 4 was divided by the total number of experts to obtain the CVI for each item. The CVI and CVR values of the measurement tool were determined to be 0.98. In the final stage of adaptation, the scale was applied to 30 nurses as a pilot study.

#### Construct validity

**Fig. 1:**
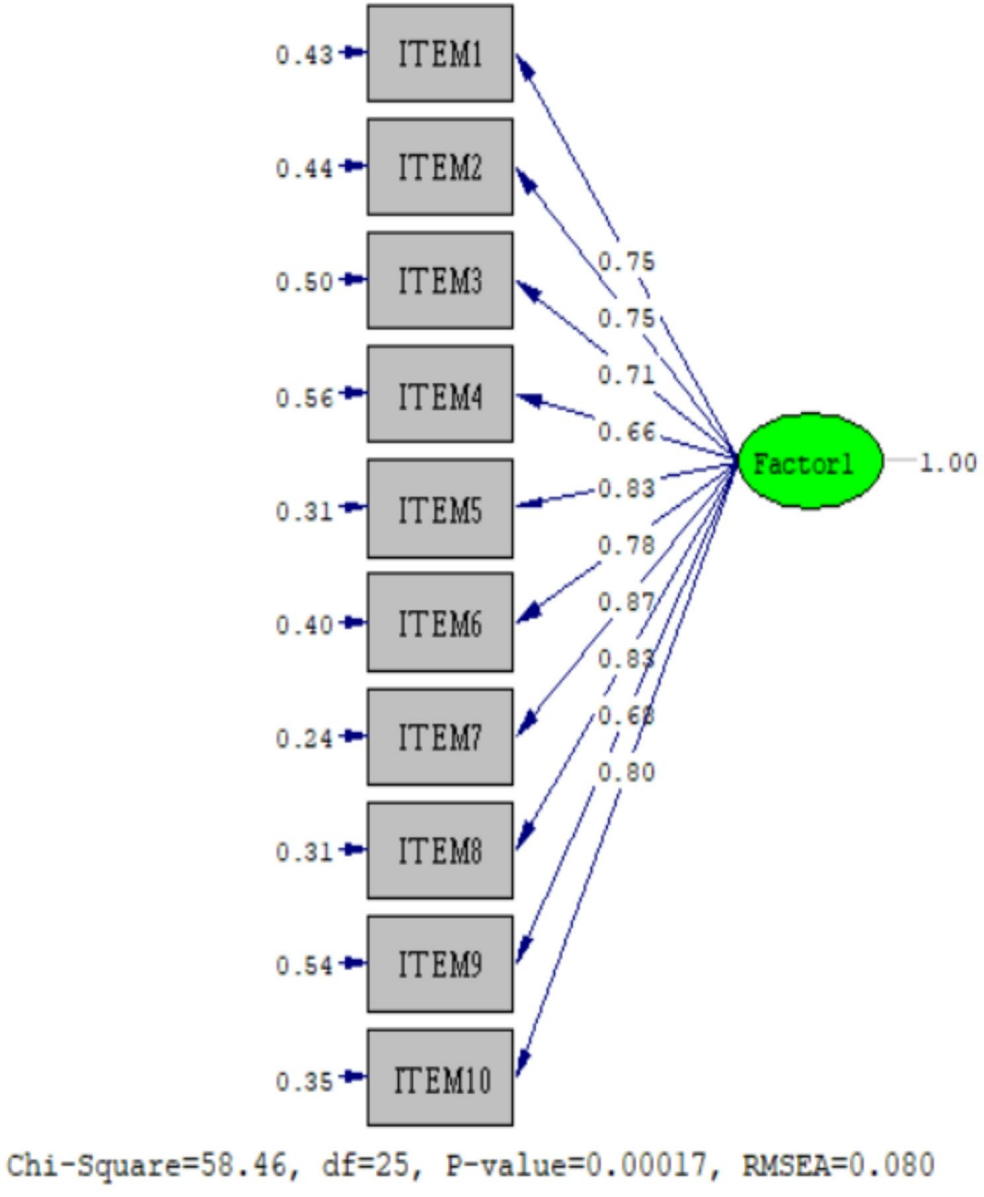
Confirmatory factor analysis of the regret intensity scale

To examine the construct validity of the scale, the results of 402 participants were randomly divided into two groups. EFA was performed with the data of 193 participants, and CFA was performed with the data of 209 participants. The results of the EFA showed a KMO index of 0.890 and a Bartlett’s global test value of 1466.942 (*p* < 0.001), confirming the suitability and adequacy of the sample. The single-factor model explained 62.9% of the variance, with factor loadings ranging from 0.737 to 0.865. The CFA performed with the data from 209 participants also supported the single-factor structure of the 10-item scale. Standardized factor loadings ranged from 0.24 to 0.56 (Table 1). The model demonstrated good fit, with χ²/df, RMSEA, GFI, AGFI, NNFI, CFI, and SRMR values presented in Table 2, and the CFA representation of the scale model is shown in Fig. 1.

**Table 1:** Item analysis results for the regret intensity scale (item-level result)

ITEMS		Mean ± SD (*n* = 402)	EFA factor loading (*n* = 193)	CFA path coefficients (*n* = 209)	α if item deleted ^1^ (*n* = 193)	α if item deleted ^2^ (*n* = 209)	α if item deleted ^3^ (*n* = 402)
1	I feel negative emotions.	3.24 ± 1.24	0.794	0.43	0.927	0.931	0.929
2	I feel uncomfortable.	3.29 ± 1.24	0.802	0.44	0.927	0.931	0.929
3	I feel worthless.	2.65 ± 1.29	0.804	0.50	0.926	0.935	0.931
4	I feel ashamed of myself.	2.38 ± 1.34	0.762	0.56	0.929	0.938	0.934
5	I feel a knot in my stomach.	2.62 ± 1.35	0.865	0.31	0.923	0.931	0.927
6	I feel anger building up inside me.	2.65 ± 1.32	0.796	0.40	0.927	0.933	0.930
7	I find it difficult to fall asleep at home.	2.76 ± 1.33	0.737	0.24	0.930	0.929	0.930
8	I have difficulty concentrating at work.	2.65 ± 1.28	0.804	0.31	0.926	0.930	0.928
9	I feel that I am no longer suitable for this job.	2.42 ± 1.32	0.748	0.54	0.930	0.936	0.933
10	I feel like crying.	2.56 ± 1.37	0.812	0.35	0.926	0.932	0.929

SD: Standard Deviation; EFA: Exploratory Factor Analysis (item-level loadings); CFA: Confirmatory Factor Analysis (standardized factor loadings and path coefficients); α: Reliability values (Cronbach’s alpha). 1: Reliability values for EFA were calculated with the data of 193 participants. 2: Reliability values for CFA were calculated with the data of 209 participants. 3: Total reliability values

**Table 2:** CFA fit index values and reference values of the scale (model fit indices)

	χ 2 / df	RMSEA	GFI	AGFI	NNFI	CFI	SRMR
Scale Fit Indexes	2.338	0.079	0.95	0.91	0.98	0.99	0.030
Reference standards	< 5	< 0.08	> 0.90	> 0.90	> 0.90	> 0.90	< 0.08

χ 2 / df: Chi-Square Test of Model Fit (Chi-Square/degree of freedom) RMSEA: root mean square error of approximation GFI: Goodness-of-Fit Index; AGFI: adjusted GFI; NNFI: Non-Normed Fit Index; CFI: Comparative Fit Index; SRMR: Standardized Root Mean Squared Residual

#### Reliability findings

The reliability of the scale was assessed using Cronbach’s alpha coefficient and the parallel form method. The total Cronbach’s alpha value obtained from the scale was 0.932 (*N* = 402; 95% CI: 0.926–0.945), and the Cronbach’s alpha if item deleted values of all items ranged between 0.927 and 0.934, indicating that the scale is highly reliable. In addition, the Cronbach’s alpha values calculated separately for the EFA and CFA subsamples are presented in Table 1.

**Fig. 2:**
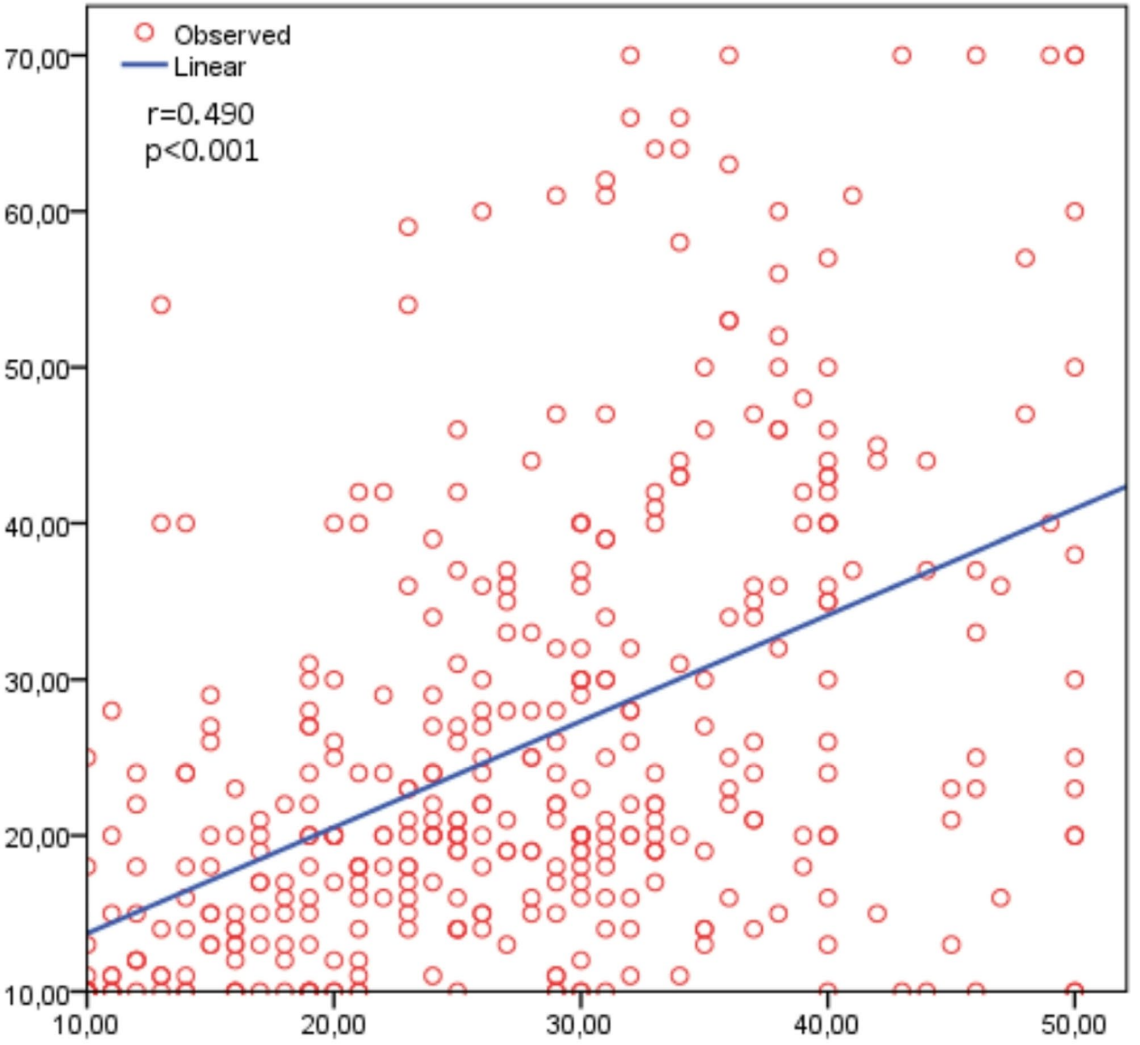
Relationship between elements of regret scale and regret intensity scale

The Elements of Regret Scale was applied as a parallel form analysis. Cronbach’s alpha was found to be 0.969 (95% CI = 0.964–0.973). The overall score was calculated by summing the 10 items of the Elements of Regret Scale and the Regret Intensity Scale; the distribution of the two scales is shown in Table 3. A statistically significant relationship was found between the two scales (*r* = 0.490; *p* < 0.001) (Fig. 2).

**Table 3:** Descriptive statistics of regret intensity scale and elements of regret scale

	Mean	Median	SD	Skewness	Kurtosis
Regret Intensity Scale	27.21	27	10.44	0.21	-0.65
Elements of Regret Scale	25.43	20	14.51	1.22	0.94

SD: Standard Deviation

## Discussion

Care-related regret refers to the regret that nurses experience due to their care decisions or actions [5]. Such feelings typically occur when a patient’s outcomes are not optimal, unexpected complications arise, or when decisions are perceived as conflicting with ethical or professional values [11]. There is a need for valid and reliable assessment tools to transform nurses’ subjective perceptions of care-related regret into objective data. For this purpose, Turkish validity and reliability analyses of the Regret Intensity Scale were performed in this study. The scale was concluded to be a valid and reliable tool in measuring nurses’ care-related regret.

Validation began with language equivalence testing. After the translation and back-translation stages, the scale items were submitted to expert opinion, and CVI was calculated, which is expected to be at least 0.80 [24]. In the study, the CVI score of the scale was found to be 0.98. In the final stage, a pilot study was conducted with 30 nurses, and feedback was received from the nurses that they had no difficulty in understanding and responding to the scale statements. Thus, no changes were required. As a result, no scale item was removed, and it was concluded that the scale items were appropriate in terms of language and content validity.

EFA is conducted at the construct validity stage in order to define the subscales of the feature to be measured [25, 26]. In the study, the KMO value was found to be 0.89, in line with the principal components analysis results, according to the EFA. A KMO coefficient close to 1 indicates the suitability of the data for analysis. Bartlett’s test is a statistical analysis method used to test whether the data come from a multivariate normal distribution. It was used to test correlations among scale items [27]. The p-value obtained (*p*< 0.0001) was statistically significant, indicating that the correlation was suitable for analysis.

CFA is used to verify the revealed structure of the scale [27]. CFA results showed factor loadings ranging from 0.737 to 0.812. In the related literature, it is recommended that scale factor loadings should be above 0.30 [26]. Accordingly, it was determined that the scale, which originally had 10 items, could be used with 10 items in the Turkish form, and all items of the scale were grouped under one factor, as in the original. The fit of the obtained data was evaluated with CMIN/df, RMSEA, CFI, AGFI, NNFI, GFI, and IFI fit criteria. When these values of the study are examined, the χ²/sd value between 2 and 3 indicates that the model shows a good fit, and the RMSEA value between 0.06 and 0.08 indicates an acceptable level of fit. GFI (≥ 0.95) and AGFI (≥ 0.90) values in the acceptable fit range indicate that the model is sufficiently compatible with the data. In addition, NNFI (≥ 0.95) and CFI (≥ 0.95) values are within the limits of a perfect fit, supporting the conclusion that the model has a good structure. The SRMR value below 0.05 indicates that the model is in a very good fit with the observed data [21]. These findings revealed that the fit indices of the scale were significant.

Cronbach’s alpha coefficient, which is obtained by dividing the total variance of the scale items by the overall variance, is used to analyze the internal consistency of the scale. The resulting value is a coefficient that indicates how closely related the items are [27]. Generally, Cronbach’s alpha value ranges from 0 to 1; a value of 0.70 or above indicates that the scale has good internal consistency. In particular, values of 0.80 and above are considered indicative of high internal consistency, and values of 0.90 and above are considered to indicate excellent internal consistency [28]. In the original study of the scale, the Cronbach’s alpha coefficient was found to be 0.87. In the German translation study by Richer et al. [6], this ratio was reported as 0.88; in the study by Neves Siderle et al. [17], it was reported as 0.86; and in the study by Ballesteros et al. [2], it was reported as 0.94. In this study, the total Cronbach’s alpha value for the Turkish version of the scale was found to be 0.93, indicating excellent internal consistency. Additionally, item-total correlation analysis was conducted as part of the reliability assessment of the scale. According to the results obtained, item-total correlation values ranged from 0.928 to 0.934. In the related literature, item-total correlation values above 0.40 are considered to indicate very good correlations [24]. In this study, item–total correlation values of 0.80 and above suggest that the items are strongly consistent with the overall construct of the scale. These findings indicate that the scale demonstrates strong internal consistency, and all items were retained without deletion.

Ballesteros et al. [2] explored the strongest triggers of regret in nurses caring for patients with multiple sclerosis (MS). In particular, the statements ‘I feel a knot in my stomach’, ‘I feel ashamed of myself’, and ‘I feel uncomfortable’ had the highest correlation with nurses’ feelings of regret. The experience of regret in MS care was directly associated with nurses’ inability to answer patients’ questions in a timely manner, failing to discuss all issues sufficiently, conveying imprecise information as if it were precise, and being concerned about misleading patients (rho >0.40, p < 0.05) [2]. In the study, the nurses had no difficulty in understanding all items, but the statement *‘I feel ashamed of myself*’ was the most difficult item to answer. These findings suggest that nurses’ regret carries not only professional but also deep psychological dimensions. In the context of Turkish nursing culture, this finding may also reflect cultural factors. Expressing emotions such as shame can be challenging due to social norms and professional expectations, as openly acknowledging shame may be perceived as a sign of weakness or inadequacy. Therefore, items involving shame may cause discomfort or hesitation among respondents, underlining the importance of considering cultural aspects when adapting psychological measurement tools.

Criterion validity is based on examining the relationship between a scale and a previously validated measurement tool to determine how accurately it assesses the concept it aims to measure [24]. In this context, the correlation coefficient (*r*) is a crucial indicator of the direction and strength of the relationship between two variables. Generally, an *r*-value above 0.30 indicates a significant relationship between the two scales, supporting the validity of the newly adapted scale [26]. In this study, a statistically significant relationship was found between the Elements of Regret Scale used as a parallel form within the scope of criterion validity and the ‘Regret Intensity Scale‘*(r* = 0.490; *p* < 0.001). This result indicates a moderate but meaningful correlation, suggesting that the new scale is consistent with other instruments measuring similar concepts and can therefore be considered a valid assessment tool.

The nurses in the present study had a moderate level of care-related regret. Previous research has shown that health professionals experience regret more intensely when they feel a greater sense of responsibility in specific situations [6]. Our findings are consistent with international studies that validate the RIS-10 in Germany [6], Portugal [18], and China [8], all of which demonstrated good psychometric properties across diverse populations. By aligning with these results, the present research contributes to the cross-cultural evidence base and underscores the international relevance of assessing care-related regret among nurses. Overall, the results suggest that the feeling of regret among nurses is not limited to a specific period of time and can occur at any stage of their professional life. Moreover, the intensity of regret may change depending on the time elapsed since the event. However, the temporal framework of the scale used is limited to the last five years, which may not fully reflect the long-term effects of this emotion [2]. Therefore, it is of great importance to administer the scale periodically to better understand the dynamic nature of the experience of regret and to comprehensively assess the emotional burden that health professionals face throughout their professional lives.

In the Turkish nursing context, the practical implications of using the RIS-10 are significant. Identifying whether nurses experience regret after providing care offers insight into the ethical dilemmas they face at the bedside, such as conflicts between professional values and clinical realities. Systematic assessment of regret can also guide the development of training programs that help nurses better express and manage their emotions. This will reduce the risk of burnout and enhance psychological well-being. Moreover, integrating regret measurement into routine practice could inform institutional policies aimed at improving communication, resource allocation, and support systems for nurses. Ultimately, the availability of a validated Turkish tool enables healthcare leaders to translate these findings into actionable strategies to strengthen both nurse well-being and patient care quality.

### Limitations of the study

This study has several limitations. First, obtaining data online may have led participants to respond with varying levels of attention. Second, the self-report design may have increased the likelihood of socially desirable responses. Third, the exclusive use of questionnaires limited the ability to explore the deeper impact of regret on individuals. In addition, restricting data collection to a specific period may have influenced participants’ responses due to contextual factors. Finally, two important psychometric aspects were not assessed: test–retest reliability and divergent validity. Moreover, cultural factors that may influence the expression of emotions, such as shame, were not systematically examined, which could affect willingness to respond and reported intensity. Future research should address these gaps to further strengthen the evaluation of the RIS-10.

## Conclusions and recommendations

This study shows that the Turkish version of the Regret Intensity Scale has good psychometric properties. The original version structure of the scale was preserved in the Turkish version. Therefore, it can be considered a valid and reliable tool for assessing the level of regret among nurses. Care-related regret is a significant emotional state that directly impacts nurses’ professional satisfaction, psychological well-being, and the quality of patient care. In this respect, measurement tools tailored to this area will play a crucial role in understanding the professional challenges faced by nurses and in developing effective supportive strategies. Testing the scale in groups of nurses working in various clinical settings and cultural contexts will both increase the generalizability of the scale and contribute to determining care-related regret levels. In addition, the results suggest that the relationship between the scale and variables such as occupational stress, burnout, and quality of patient care should be examined in greater depth. Future studies can investigate the change in care-related regret in nurses over time and its effect on professional decision-making processes with the long-term use of the scale. Furthermore, cross-validation of the scale in more diverse nursing populations (e.g., community nurses, nurses working in private hospitals) is recommended, as well as longitudinal studies that will allow the evaluation of changes in care-related regret over time. In this context, it is recommended to evaluate the levels of care-related regret in other professionals in the field of health by applying the scale to different professional groups.

## Data Availability

Data are available from the corresponding author on reasonable request.
